# Effect of fetoscopic laser surgery on the placental characteristics and birth-weight discordance of twins with twin-to-twin transfusion syndrome

**DOI:** 10.3389/fmed.2022.942816

**Published:** 2022-09-29

**Authors:** Xueju Wang, Luyao Li, Pengbo Yuan, Yangyu Zhao, Yuan Wei

**Affiliations:** Department of Obstetrics and Gynecology, Peking University Third Hospital, Beijing, China

**Keywords:** twin-to-twin transfusion syndrome, birth-weight, placental territory, anastomoses, fetoscopic laser surgery

## Abstract

**Objective:**

This study explored the effect of fetoscopic laser surgery on the placental structure and birth-weight discordance of twin-to-twin transfusion syndrome (TTTS).

**Methods:**

A retrospective cohort study was conducted in TTTS patients who were admitted to the Peking University Third Hospital between April 2014 and April 2020. The patients were divided into two groups: laser group and control group. Placentas with twin survival were injected, and pregnancy outcomes and placental characteristics of the two groups were compared. The correlation between the birth-weight discordance and placental characteristics in each group was analyzed.

**Results:**

The gestational age at first diagnosis in the laser group was significantly smaller than that in the control group (21.6 ± 2.8 weeks vs. 27.7 ± 3.0 weeks, *p* < 0.001). The proportion of patients with TTTS stage-I in the laser group was significantly lower than the control group (9.4 vs. 64.0%, *p* < 0.001). The gestational age at delivery in the laser group was significantly larger than that in the control group (33.6 ± 2.1 weeks vs. 31.4 ± 2.5 weeks, *p* = 0.001). In the laser group, the birth-weight discordance ratio was positively correlated with the placental territory discordance ratio (Spearman coefficient = 0.556; *p* = 0.001).

**Conclusion:**

The birth-weight discordance is positively correlated with placental territory discordance in TTTS patients after FLS.

## Background

Twin-to-twin transfusion syndrome (TTTS), as a specific complication of monochorionic twins, accounts for 10–15% of monochorionic twin pregnancies (1, 2). Previous studies have demonstrated that the placental characteristics, in addition to placental anastomoses, placental share, and umbilical cord insertion sites affect the treatment and prognosis of TTTS. Although the mechanism involved in the initiation of blood transfusion between two fetuses remains elusive, and it is currently considered that the superficial artery-vein (AV) anastomoses of the monochorionic placenta serve as the anatomical basis of TTTS (3). Therefore, the first-line treatment for TTTS is fetoscopic laser surgery (FLS) that causes superficial placental anastomoses to coagulate, thus blocking the blood flow and exchange of blood between the two fetuses. Previous studies have confirmed that FLS can significantly reduce the incidences of intrauterine fetal death and neonatal mortality of TTTS and improve the pregnancy outcome (4). However, no related studies have been published on the birth-weight discordance of two neonates in TTTS patients after FLS; therefore, the influencing factors remain to be ascertained. As a complex twin pregnancy resulting from the special placenta of monochorionic twins, its pathogenesis, diagnosis, treatment, and prognosis, with due consideration to the structure of the placenta, need to be discussed (3, 5–7). This study attempted to compare the placental characteristics of TTTS with conservative treatment and FLS treatment as well as to explore the correlation between the placental characteristics and the birth-weight discordance of two neonates in TTTS patients after FLS treatment.

## Methods

This study was based on a retrospective analysis of the TTTS patients admitted to Peking University Third Hospital between April 2014 and April 2020. The patients who chose to terminate the pregnancy out of the fear of the negative neonatal outcome, those who delivered the baby/ies at the local hospital, those who had one or two fetuses intrauterine death, and those with placenta breakage after the delivery failed to perfusion were excluded from the study. The remaining patients with two live neonates were divided into the control and laser treatment groups. The control group included patients in whom the operation could not be performed because of the factors such as the gestational age, patients who underwent conservative treatment at the TTTS Stage I, or those patients received reduction in the amniotic fluid. The laser-treatment group included patients who received FLS treatment. The approval of the ethics committee of the hospital was sought for procuring the placenta of the patients after delivery with their informed consent, on which perfusion was performed later. To investigate the correlation between the birth-weight discordance and placental characteristics in each group, the placentas of TTTS patients who delivered live twins were examined.

TTTS diagnostic criteria: Ultrasound examination of women with monochorionic twin pregnancies based on the polyhydramnios-oligohydramnios sequence (8), which means that, before 20 weeks, the maximum vertical pocket (MVP) of the recipient fetus was ≥ 8 cm, while the MVP of the donor fetus was ≤ 2 cm. After 20 weeks, the MVP of the recipient fetus was ≥ 10 cm, while the MVP of the donor fetus was ≤ 2 cm. According to the Quintero staging criteria (8), TTTS was divided into five stages, as follows: stage I: the bladder of the donor fetus is visible; stage II: the bladder of the donor fetus is no longer visible; stage III: the presence of any fetus showing abnormal blood flow; stage IV: the recipient fetus showing edema; and stage V: any or all fetuses are dead.

After seeking approval of the ethics committee and the medical record department, we employed the medical record system to collect the clinical data included the age of the patients, complications associated with their pregnancy, the gestational age when TTTS was diagnosed, therapeutic regime, the gestational age of the fetus at the time of delivery, and the birth weight of the two neonates. Birth weight discordance ratio = (weight of heavier neonate – weight of lighter neonate)/weight of heavier neonate.

**FIGURE 1:**
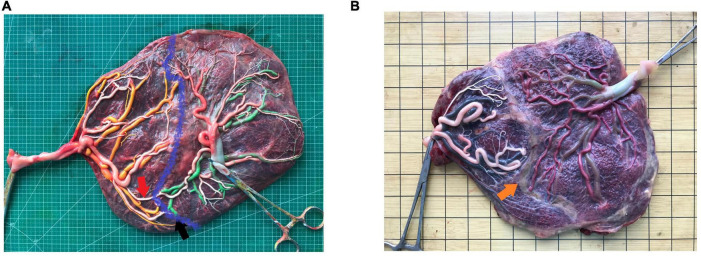
An image of the placenta after dye injection **(A)** placenta without laser surgery, red arrow, artery-artery anastomosis, black arrow, artery-vein anastomosis, blue curve, placental separate line; **(B)** placental with laser surgery, orange arrow, laser coagulation line.

The placenta of the patients with monochorionic twins after delivery were examined in the hospital to verify the diagnosis. According to the protocol published by our center and past studies (9–11), all intact monochorionic placentas were perfused with the pigment to examine the placental anastomoses, placental portions, and insertion of the umbilical cord (Figure 1A). Briefly, after delivery, the amniotic membranes were removed, and each umbilical cord was cut 5 cm from its placental insertion site. The placental vessels were gently squeezed to eliminate the blood clots. The umbilical vein and one of the umbilical arteries were then cannulated and clamped with an intravenous catheter. The placental vessels were injected with saline until all the branches were visible. The last step was performed using four 20-mL syringes, each filled with a distinctively colored dye (i.e., white, green, yellow, and red) to visualize the umbilical cord arteries and veins of the two fetuses. Images of the injected placentas were placed vertically under a grid harboring a scale were captured with a high-resolution digital camera.

We measured and recorded the features indicating superficial placenta that included the type, number, diameter of anastomoses, the placental share between the two fetuses, and the distance between the umbilical cord insertion points. The diameter of artery–artery (AA) and vein–vein (VV) anastomoses was measured in the narrowest part. Deep arterio-venous (AV) anastomoses were defined as sites where an unpaired artery from one twin had penetrated the chorionic plate within < 1.0 mm of an unpaired vein from the other twin and the diameter of AV anastomoses was measured on the arterial side (10). The anastomoses of the placenta after the FLS operation were residual anastomoses (Figure 1B). The attachment sites of the umbilical cord included marginal attachment, velamentous attachment, and central attachment. To avoid the influence of individual differences on the size of the placenta, the umbilical cord insertion ratio was determined, that is, the distance between two umbilical cord insertion points/maximum placental diameter. The umbilical cord insertion distance was defined as the distance between the centers of two umbilical cord insertion sites (9–11). The maximum placental diameter was the largest range of placental parenchyma edges (10). The placental territory of the two fetuses was determined on the basis of the sites of anastomoses and was measured using the ImageJ 1.51j8 software for windows (National Institute of Health, USA). The placental territory discordance ratio = (large placental territory – small placental territory)/large placental territory.

The SPSS 24.0 software was used to analyze the data. The data were expressed as mean ± standard deviation or median (maximum, minimum). The data was analyzed to determine whether it was normally distributed. If the data was found to be normally distributed, an independent sample *t*-test and Pearson’s correlational analysis were performed, and the mean ± standard deviation was used as the statistical measure to express the data. In case the data did not show normal distribution, Mann–Whitney test, a non-parametric test, and Spearman correlational analysis were performed, and the median and range were used for expressing the data. Categorical data were compared using Chi-square test or Fisher’s exact test, as deemed appropriate. *P* < 0.05 was considered to be statistically significant.

## Results

**FIGURE 2:**
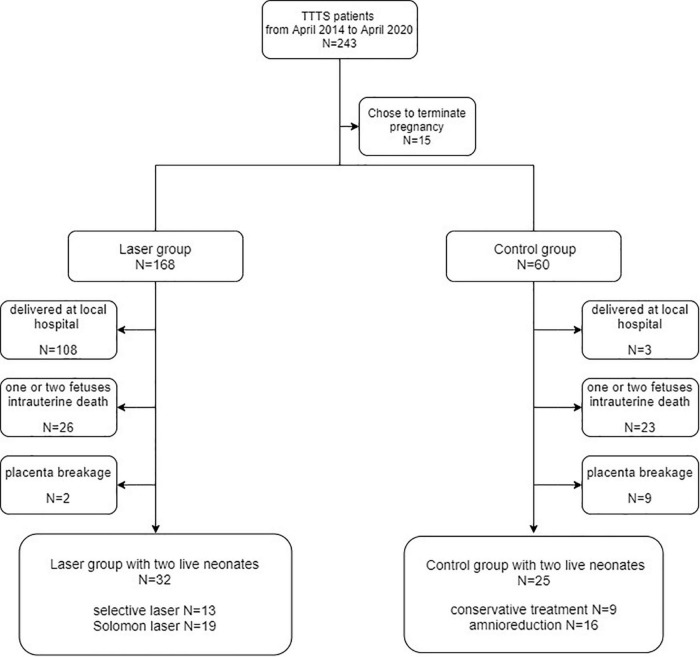
Flowcharts depicting the patient inclusion criteria.

A total of 243 TTTS patients were admitted to the Obstetrics Department of Peking University Third Hospital from April 2014 to April 2020, among whom 15 patients opted to terminate their pregnancies. Moreover, 168 patients were treated with laser and 60 underwent a conservative treatment, therapeutic amniocentesis. The patients who delivered the baby/ies at other hospitals, those who had one or two fetuses intrauterine death, and those with placenta breakage after the delivery failed to perfusion were excluded from the study. Retrospective analysis of the data of 57 TTTS patients, including 32 patients in the laser group and 25 patients in the control group, was performed. Figure 2 illustrates the details regarding the patients included in the study and the criteria for inclusion.

**C**omparison of the clinical data of the two groups of TTTS patients has been illustrated in Table 1. The gestational age at the preliminary diagnosis of TTTS in the laser treatment group was significantly lower than that in the control group. The difference between the two groups with respect to the Quintero stages was statistically significant. The comparison of the two groups revealed that the proportion of stage I in the laser treatment group was significantly lower than that in the control group (*P* < 0.001) and that there was no significant difference between the two groups with regard to the respective stages: Stage II, III, and IV (*P* = 0.052/0.064/1.000, respectively). The gestational age at the time of delivery, the birth weight of the recipient fetus, and the birth weight of the donor fetus in the laser treatment group were significantly higher than those in the control group.

**TABLE 1:** The clinical features of TTTS patients between the two study groups.

Category	Laser treatment (*n* = 32)	Control (*n* = 25)	*P*-value
Age [Year, *M* (*min–max*)]	27 (24–38)	29 (23–40)	0.382
Application of assisted reproductive technology [Case (%)]	4 (12.5)	1 (4.0)	0.372
Hypertensive disorders in pregnancy [Case (%)]	2 (6.3)	1 (4.0)	1.000
Gestational diabetes [Case (%)]	4 (12.5)	2 (8.0)	0.686
The gestational age at the first diagnosis of TTTS (±*s*)	21.6 ± 2.8	27.7 ± 3.0	< 0.001
**Quintero stage [Case (%)]**			
Stage I	3 (9.4)	16 (64.0)	< 0.001
Stage II	12 (37.5)	2 (8.0)	0.052
Stage III	10 (31.3)	1 (4.0)	0.064
Stage IV	7 (21.9)	6 (24.0)	1.000
Gestational age of delivery	33.6 ± 2.1	31.4 ± 2.5	0.001
The birth weight of the recipient fetus (g)	2076 ± 438	1792 ± 599	0.044
Neonatal asphyxia [Case (%)]	5 (15.6)	6 (24.0)	0.508
The birth weight of the donor fetus (g)	1645 ± 477	1334 ± 393	0.011
Neonatal asphyxia [Case (%)]	4 (12.5)	9 (36.0)	0.056
Birth weight discordance ratio	0.23 (0.01, 0.61)	0.24 (0.01, 0.57)	0.426

*M* (*min–max*) represent the median (minimum–maximum).

The placental characteristics of the patients in the two groups have been illustrated in Table 2. The correlations between birth weight discordance ratio and the total diameter of AA anastomoses in the superficial placenta, the total diameter of AV anastomoses, the total diameter of VV anastomoses, placental territory discordance ratio, and umbilical cord insertion ratio in each group were analyzed respectively in Table 3. The results demonstrated that the birth weight discordance ratio was positively correlated with the placental territory discordance ratio in the laser treatment group (Spearman coefficient = 0.556). There was no positive linear correlation between the birth-weight discordance ratio and the indicators of placental structure in the control group.

**TABLE 2:** The placental characteristics of TTTS patients between the two study groups.

Category	Laser treatment (*n* = 32)	Control (*n* = 25)	*P*-value
AA anastomoses*			
The prevalence rate [Case (%)]	7 (21.9)	8 (32.0)	0.546
The total number [Count, *M* (min–max)]	1 (1,1)	1 (1,1)	1.000
The total diameter [mm, *M* (min–max)]	3.6 (0.7,5.1)	2.0 (0.5,5.3)	0.094
AV anastomoses*			
The prevalence rate [Case (%)]	9 (28.1)	24 (96.0)	< 0.001
The total number [Count, *M* (min–max)]	4 (1,9)	5 (1,13)	0.207
The total diameter [mm, *M* (min–max)]	2.1 (1.1,7.4)	5.5 (1.3,15.3)	0.018
VV anastomoses*			
The prevalence rate [Case (%)]	9 (28.1)	6 (24.0)	0.771
The total number [Count, *M* (min–max)]	1 (1, 1)	1 (1,2)	0.607
The total diameter [mm, *M* (min–max)]	3.0 (0.7,6.4)	1.6 (1.0,6.5)	0.955
The total number of all types of anastomoses*	0 (0,11)	6 (2,14)	< 0.001
The total diameter of all types of anastomoses*	0 (0,16.8)	6.1 (1.7,18.6)	< 0.001
Placental territory discordance ratio [*M* (min–max)]	0.39 (0.02,0.67)	0.25 (0.02,0.75)	0.236
Velamentous umbilical insertion [Case (%)]	6 (18.8)	3 (12.0)	0.717
Umbilical cord insertion ratio	0.61 ± 0.20	0.66 ± 0.17	0.377
Birth weight discordant ratio/placental territory ratio	1.55 (0.17,17.29)	1.26 (0.03,15.17)	0.074

*M* (*min–max*) represent the median (minimum–maximum), *Residual vascular anastomosis for laser-treatment group.

**TABLE 3:** Correlation between birth-weight discordance ratio and the placental structure characteristics in the two study groups.

			The total diameter of AA anastomoses [mm, *M* (min–max)]	The total diameter of AV anastomoses [mm, *M* (min–max)]	The total diameter of VV anastomoses [mm, *M* (min–max)]	Placental territory discordance ratio	Distance ratio of umbilical cordattachment points
	Laser treatment group 0.23 (0.01,0.61)	Figures	3.6 (0.7,5.1)	2.1 (1.1,7.4)	3.0 (0.7,6.4)	0.39 (0.02,0.67)	0.62 (0.15,1.00)
Birth weight discordance ratio		Spearman coefficient	−0.468	0.248	−0.067	0.556	−0.250
		*P*-value	0.289	0.171	0.865	0.001	0.167
	Control group 0.24 (0.01,0.57)	Figures	2.0 (0.5,5.3)	5.5 (1.3,15.3)	1.6 (1.0,6.5)	0.25 (0.02,0.75)	0.65 (0.38,1.00)
		Spearman coefficient	0.551	−0.085	0.314	0.329	0.099
		*P*-value	0.157	0.686	0.544	0.108	0.638

*M* (*min–max*) represent the median (minimum–maximum).

## Discussion

The pathogenesis of TTTS is closely associated with the placental structure (7). Previous studies have confirmed that the shared placenta, vascular anastomoses, and umbilical cord attachment are essential factors that affect the incidence of complex complications and prognosis in monochorionic twins (5, 12–15). Therefore, complex twins are considered a placental disease. First, the study explored the effect of fetoscopic laser surgery on the placental characteristics and birth-weight discordance of twins with twin-to-twin transfusion syndrome.

The findings of the present study revealed that, when compared to the control group, a significant positive correlation was recorded between birth weight discordance ratio and placental territory discordance ratio in the laser treatment group (Spearman coefficient = 0.556). Lewi et al. conducted a study of 100 cases of monochorionic twin placentas without TTTS and reported that the birth weight discordance ratio was positively correlated with the placental territory discordance ratio (16). Considering that the TTTS patients were excluded from the study conducted by Lewi et al. the factors influencing the birth weight discordance in the two live births by TTTS patients, especially in those who received FLS, remained elusive. Birth weight discordance was found to be positively correlated with placental territory discordance in TTTS patients who received FLS. The findings of the present study were consistent with that of the study conducted by Lewi et al. Moreover, our study focused on TTTS patients and thus compensated for the limitations of the research conducted by Lewi et al. On the basis of these findings, it was speculated that when placental anastomoses were coagulated with successful FLS, placental sharing between the two fetuses impacted their growth. Therefore, neonatal birth weight discordance was positively correlated with placental territory discordance. The placental territory of the donor fetus is always smaller than that of the recipient fetus (17), as anastomoses from the recipient fetus to the donor fetus might compensate for the blood supply of the smaller placental territory to a certain extent in TTTS patients. Coagulation of anastomoses after fetoscopic surgery aggravates the degree of ischemia and hypoxia in the fetus with a smaller placental territory (16).

Previous studies have reported an incidence of selective intrauterine growth restriction (sIUGR) in patients with TTTS of 40–78% (18), and the incidence of intrauterine death of the fetus with growth restriction was higher in patients after FLS (18, 19). A recent study conducted in our center found that the occurrence of thick placental AA anastomoses in TTTS with sIUGR was significantly higher than in those with only TTTS (20). The thick AA anastomoses could partially supplement the blood supply to the growth-restricted fetus. It was thus speculated that the coagulation of these thick AA anastomoses by FLS aggravates the degree of ischemia and hypoxia in fetuses suffering from restricted growth, which led to intrauterine death of the fetuses. However, the fetoscopic surgery creates a distinct separation of the two fetal vascular territories on the surface of the placenta (4). FLS can significantly improve the pregnancy and neonatal outcome of TTTS, which is consistent with our results and the finding of relevant past studies. Placental characteristics affect the prognosis of TTTS. The lower prevalence of AA anastomoses could potentially lead to these conflicting outcomes (21). TTTS is caused by unbalanced vascular anastomoses within the placenta. AV anastomosis is believed to act as the foundation of TTTS. AA anastomosis is believed to act as a protective factor against TTTS occurrence (21, 22). There was at least one AV anastomosis on almost all TTTS placentas, whereas past studies have revealed a low prevalence of AA anastomoses in the TTTS placentas (3). The incidence of AV anastomoses was 95.8%, while that of AA anastomoses was 33.3% (11). Therefore, it was suggested that further study should be undertaken to explore whether the thick AA anastomoses of TTTS patients with sIUGR should be preserved during FLS.

For the indication and timing of FLS in TTTS patients, most studies suggested that 16–26 weeks were suitable for FLS of TTTS patients in Stages II–IV (2). Our study indicated that the proportion of patients in Stage I in the laser treatment group was significantly lower than that in the control group and that the gestational age of the fetus in the laser treatment group at the time when the disease was first diagnosed was lower than that in the control group. This finding was consistent with the findings of the studies conducted earlier. Numerous studies have confirmed that FLS can significantly improve the pregnancy outcome of TTTS patients (4). The results of the present study also found that the gestational age at the time of delivery, the birth weight of the recipient fetus, and the birth weight of the donor fetus in the laser treatment group were significantly higher than those in the control group, which is consistent with the finding of the studies conducted earlier (4).

The study also posed some limitations. First, most of the patients returned to the local hospital for delivery after undergoing FLS in our hospital. Second, the sample size of this study was relatively small and hence the probability of statistical bias could not be ignored. Third, because of a significant time span, two different methods of FLS, namely selective laser coagulation and Solomon operation were performed during this period in our hospital. As the effects of the two methods could not be compared, considering the small sample size, the possibility of statistical deviation could not be ignored.

## Conclusion

In summary, the findings of the present study indicated that birth weight discordance between two live births was positively correlated with the placental territory discordance in TTTS patients after FLS.

## Data availability statement

The raw data supporting the conclusions of this article will be made available by the authors, without undue reservation.

## Ethics statement

The studies involving human participants were reviewed and approved by the Peking University Third Hospital. The patients/participants provided their written informed consent to participate in this study.

## Author contributions

XW and LL conceived the study concept and wrote the manuscript. LL and PY collected the data and contributed to data analysis. YZ contributed to data consultation and study guidance. YW contributed to manuscript revision. All authors contributed to the article and approved the submitted version.

## Abbreviations

FLS: fetoscopic laser surgery
TTTS: twin-to-twin transfusion syndrome
AA: artery-to-artery
AV: artery-to-vein
VV: vein-to-vein.
